# Development study of scientific competence and activity scale in medical faculty research assistants

**DOI:** 10.55730/1300-0144.6026

**Published:** 2025-05-19

**Authors:** Cemal KOÇAK, Hande GÜVERCİN, Ecem ESEN, Meltem ÇÖL

**Affiliations:** Department of Public Health, Faculty of Medicine, Ankara University, Ankara, Turkiye

**Keywords:** Medical faculty research assistants, evidence-based medicine, research knowledge and skills, academic life, the scientific competence and activity scale

## Abstract

**Background/aim:**

One mission of medical faculties is training scientists equipped with the necessary knowledge, skills, and attitudes, who can evaluate the scientific research. The aim was to develop a scale for evaluating the level of scientific competence and activity in research assistants.

**Materials and methods:**

The study is a methodological study, conducted in October 2023–May 2024, with 299 research assistants working at Ankara University Faculty of Medicine. The reliability was assessed by item-total correlation and Cronbach-alpha coefficient; construct validity was assessed by EFA. In EFA, principal component analysis, Varimax rotation were used. KMO and Bartlett test p-value were calculated. Groups with eigenvalues greater than one were determined as factors. The ability of the scale score to determine the level of scientific competence and activity was examined by ROC curve analysis. SPSS 30.0 was used; significance was taken as p < 0.05.

**Results:**

KMO value is 0.945, Bartlett’s test p is <0.001. Seven items were removed. As a result of Varimax rotation, four sub-dimensions were determined; factor 1 (fourteen items), factor 2 (eight items), factor 3 (four items), factor 4 (three items). Cronbach’s alpha coefficient is 0.960, item-total correlation coefficients are greater than 0.3. The mean score was 91.23 ± 23.10. According to the ROC analysis for participation in at least one publication/project, the cut-off point was 92.

**Conclusion:**

The Scientific Competence and Activity Scale consists of 29 items in 4 subdimensions, it is valid, reliable. To generalize its validity and reliability, it is crucial to test the theoretical structure of the scale in various groups.

## 1. Introduction

The roles of universities in the context of global competition are becoming increasingly significant, leading to heightened expectations [1]. Accordingly, the rankings of universities in global league tables are constantly scrutinized, and efforts are made to improve their positions. Ranking systems focus heavily on the concept of scientific productivity, prompting the development of tools to monitor performance indicators [2]. Academics play a critical role in generating knowledge through their scientific activities and in ensuring the efficient utilization of human resources [3]. Engaging in scientific activities is also essential for academics to advance in their careers (e.g., associate professorship, full professorship) [4]. The outputs of these activities-such as articles, conference papers, and books-are published to contribute to the scientific community. The quality and originality of these outputs are regarded as indicators of the scholar’s and their institution’s contributions to the scientific world [5].

However, there are several circumstances that affect the scientific activities of academics. According to a study conducted with research assistants from different universities and departments, 75% of participants stated that being occupied with noneducational activities negatively impacts their career development, while 86% pointed to unclear job descriptions as a significant barrier [6]. In addition, in different studies have identified various obstacles to research assistants’ scientific work, including the heavy workload of departmental tasks, lack of time, poor working conditions, physical and financial constraints, limited access to information, low-quality education, insufficient academic incentive payments, and the absence of an environment fostering collaboration and solidarity [6,7].

The primary mission of medical schools is to train physicians equipped with the knowledge, skills, and attitudes required for good medical practice, capable of applying the most effective and up-to-date treatment methods, academically advanced, and engaged in scientific research. Additionally, they aim to provide high-quality healthcare services [8–10]. In recent years, there has been a greater emphasis on “evidence-based” approaches in medical practice. Evidence-based medicine involves the process of reviewing, assessing, and applying current research findings to guide clinical decisions [11]. Promoting evidence-based medicine helps foster lifelong learning skills and develop critical thinking abilities [12].

A specialty student who is knowledgeable about research principles can conduct well-designed, high-quality studies that contribute to the advancement of medicine [13]. Conducting scientific research requires adherence to specific rules, including accurately interpreting the literature and mastering research methodologies. Encouraging research assistants to engage in research from the early stages of their training enables them to stay updated with innovations and apply effective, modern treatment methods [8,9].

Despite these aspects, there is currently no scale in Türkiye to measure the scientific competence, activity, and literacy of research assistants. Assessing the scientific competence and activity levels of research assistants will provide guidance for future intervention studies and enhance the quality of research assistant training.

This study aims to develop a scale to evaluate the scientific competence and activity levels of research assistants. The Scientific Competence and Activity Scale, developed within the scope of this study, is expected to serve as a reference for assessing the competence levels of research assistants in the future. It is anticipated that the scale will contribute to enhancing research competence, ensuring high quality, and addressing and resolving issues in the field of scientific activity.

## 2. Materials and methods

### 2.1. Type of study, population, and sample

This study is a methodological scale development study conducted between October 2023 and May 2024 with research assistants from the Faculty of Medicine. According to information obtained from the Dean’s Office, the total number of research assistants working at Ankara University Faculty of Medicine was 1234. Since there is no prevalence data in the literature regarding the scientific competence and activity levels of medical faculty research assistants, the prevalence was assumed to be 50%. The sample size was calculated using the sample size formula for a known population in the Epi-Info 7.2.6 program, with a population of 1234, a 95% confidence interval, a 50% prevalence, a 5% margin of error, and a design effect of 1, resulting in a sample size of 294. Additionally, in scale development studies, it is recommended that the sample size should be at least five times the number of scale items (36 items × 5 = 180 participants), a criterion that was also met [14].

The study included 300 research assistants who agreed to participate in the study and whose written consents were obtained and distributed proportionally according to the departments. However, data from one participant were excluded due to carelessly completed survey and scale responses, leaving 299 participants for the analyses.

### 2.2. Item pool creation

National and international studies on scientific research processes were examined and the expressions that could be used in the scale were determined. Following an extensive literature review, an item pool was created for the draft version of the “Scientific Competence and Activity Scale (SCAS) for Research Assistants in Medical Faculties.” The draft scale consisted of 41 items related to scientific competence and activities, designed in a 5-point Likert format. Responses to the scale were planned as follows: “Strongly Disagree” (1 point), “Disagree” (2 points), “Neutral” (3 points), “Agree” (4 points), and “Strongly Agree” (5 points). The scale did not include any reverse-scored items. As the score on the scale increases, the level of scientific competence and activity also increases; conversely, lower scores indicate a lower level of competence and activity.

### 2.3. Expert review

During the content validity phase, opinions of 7 experts, including 2 epidemiology expert, 2 public health experts, 1 general internal medicine expert, 1 general surgery expert, 1 medical biochemistry expert, were sought to determine whether each item was adequate and appropriate in terms of content and quality [15]. To this end, the draft scale form, consisting of 41 items, was prepared with three response options; “appropriate,” “partially appropriate,” and “not appropriate” along with spaces for experts to provide written feedback for each item.

Four researchers, along with seven external academics, evaluated the scale in terms of face and content validity. Some items were deemed unsuitable for the scale or found to overlap with others. Consequently, items that were closely related, difficult to understand, not aligned with the theoretical framework, or deemed unnecessary were removed from the draft scale.

The Davis method was used to statistically analyze the expert opinions. In this method, content validity indices (CVI) were calculated for each item. These indices were determined by taking one less than the ratio of the total number of experts who answered “appropriate” for each item to half of the total number of experts. Items with a content validity index below 0.80 were excluded from the study [16]. In this context, the items with an index value of 0.43 are “I have a sufficient level of foreign language skills to write my study”, “There is a sufficient culture of scientific activity in my institution”. The items with an index value of 0.71 are “I am capable of systematically gathering the data required for research”, “I am knowledgeable about the characteristics of systematic reviews and meta-analyses”, “There are people I can consult and who can encourage me when engaging in scientific activities”. These 5 items were removed from the draft scale. The other items were deemed necessary by the experts and remained in the scale with a full indice value of 1.0.

A pilot study was conducted with 15 research assistants from various departments (internal/surgical/basic medical sciences) who were not part of the main study group. The pilot involved face-to-face interviews lasting 15–20 min. Feedback was collected regarding the clarity and ease of responding to each item, the logical sequence of items, the time required to complete the questionnaire, and any missing topics. No items were removed from the scale as a result of the pilot study, and the final version of the scale consisted of 36 items.

### 2.4. Data collection

The research team approached the research assistants, explained the study details, and shared the Google Forms link for completing the survey and scale forms. Participants were asked to fill out a consent form within Google Forms before proceeding to the data collection forms. All participants provided their consent.

The data were collected using a 15-item survey form and a 36-item Scientific Competence and Activity Scale. The survey form inquired about the sociodemographic characteristics of research assistants, their participation in scientific activities, and the presence and quality of activities such as seminars, literature reviews, and case presentations within their departments. Following the survey, the developed scale was administered. The dependent variable of the study is the scientific competence and activity score determined by the scale, while the independent variables include age, gender, marital status, number of children, department of work, duration of residency, and the number of scientific publications, conferences/workshops attended, posters presented, oral presentations delivered, and research projects conducted. Additionally, the presence and quality of departmental activities such as literature reviews, seminars, and case presentations were considered independent variables.

### 2.5. Ethical considerations

Ethical approval for this study was granted by the Ethics Committee of Ankara University’s Faculty of Medicine. Participation was voluntary, and both verbal and written consent were acquired from all participants.

### 2.6. Statistical analysis

Data analysis was conducted using the SPSS (Statistical Package for Social Sciences) version 30 software. Descriptive statistics were presented as frequency, percentage, mean, standard deviation, median, and minimum-maximum values. Chi-square tests were used to compare categorical variables. For continuous variables, normality was assessed using the Kolmogorov-Smirnov and Shapiro-Wilk tests. Since the data did not follow a normal distribution, nonparametric tests (Mann-Whitney U and Kruskal-Wallis test) were used for comparisons. The relationships between variables were evaluated using the Spearman correlation test.

#### 2.6.1. Exploratory factor analysis (EFA)

Exploratory factor analysis (EFA) was performed to assess the construct validity of the scale. For EFA, principal component analysis with Varimax rotation was applied. The Kaiser-Meyer-Olkin (KMO) value and the p-value of Bartlett’s test of sphericity were calculated to assess the adequacy of the sample for factor analysis. A KMO value above 0.5 and a p-value for Bartlett’s test below 0.05 were regarded as acceptable. The variance explained by each factor and the total explained variance were calculated. The number of factors was determined based on the criterion that factors with eigenvalues greater than 1 were retained [17]. Factor loadings had to be greater than 0.4, and items with factor loadings differing by 0.1 or less across multiple factors were removed from the analysis [18]. Items were removed iteratively, and after each removal, factor loadings were re-evaluated, and the factor analysis process was repeated [19]. Through this stepwise removal process, the most suitable scale was finalized. The ability of the scale score to determine scientific competence and activity levels was examined using Receiver Operating Characteristics (ROC) curve analysis. Once significant threshold values were determined, their sensitivity and specificity were computed. Statistical significance was defined as p < 0.05.

#### 2.6.2. Reliability assessment stage

To evaluate the reliability of the scale, the item-total score correlation and internal consistency coefficient (Cronbach’s alpha) were computed. A Cronbach’s alpha reliability coefficient of 0.7 or above and an item-total correlation above 0.3 were considered acceptable. Additionally, changes in the reliability coefficient when an item was deleted were evaluated.

## 3. Results

The average age of the 299 research assistants in the study group was 28.8 ± 3.08 years (range: 24–48). Of the participants, 58.2% were women, 55.8% were single, 0.7% were divorced/widowed, and 43.5% were married. The percentage of those who have children is 11.7%. The distribution of residency duration was as follows: 25.4% had been assistants for 0–12 months, 17.7% for 1–2 years, and 56.9% for more than 2 years. Among the participants, 65.9% (197 individuals) were from internal medical sciences, 28.8% (86 individuals) were from surgical medical sciences, and 5.3% (16 individuals) were from basic medical sciences. Additionally, 90.3% (270 individuals) were main specialty research assistants, while 9.7% (29 individuals) were subspecialty research assistants.

The frequency of being single was significantly higher in the surgical group compared to others (p = 0.014). Research assistants in surgical branches had attended more international conference, symposium, and workshop than those in internal medical sciences (p = 0.044). Internal medical sciences had significantly fewer oral presentations compared to other branches (p = 0.005). Research assistants in basic medical sciences perceived the quality (p = 0.002) of departmental activities such as seminar, literature review, and case presentation as more adequate. No significant differences were observed among the branches in terms of other variables.

Among subspecialty research assistants, compared to main specialty research assistants, there were significantly higher frequencies of being aged 30 or older, being married, having children, conducting original research, publishing case report and book chapter, attending national and international conference, presenting poster and oral presentation, and engaging in national and international research project (Table 1).

Among all research assistants from various branches, 52.5% reported having a sufficient level of foreign language proficiency to follow scientific publications (According to an item of the scale).

### 3.1. Construct validity analysis of the scale (exploratory factor analysis)

To assess validity, the KMO value was computed, and Bartlett’s test of sphericity was performed. According to Table 2, the KMO value was 0.945, and the p-value for Bartlett’s test of sphericity was <0.001. As a result of the principal component analysis, it was observed that the factor loading differences for seven items under multiple factors were 0.1 or less. These items were sequentially removed, and factor analysis was repeated. After excluding these items, the factor analysis was finalized with 29 items. The excluded items are presented in Table 2.

The scale was found to explain a total variance of 68.0%. All items forming the factors had the highest factor loadings above 0.4, ranging between 0.558 and 0.834. As a result of the Varimax rotation, it was identified that the scale consists of four subdimensions. Table 3 shows the highest factor loadings of the items under each factor. According to this, Factor 1 consists of fourteen items, Factor 2 of eight items, Factor 3 of four items, and Factor 4 of three items. Based on the common characteristics of the items representing each factor, the factors were named as follows: “Competence”, “Literacy”, “Opportunities”, “Barriers”.

### 3.2. Reliability analysis of the scale

After completing the validity analyses, the next step the purpose was to evaluate the reliability and internal consistency. The Cronbach’s alpha coefficient for the 29 items was calculated as 0.960, and the item-total correlation coefficients were observed to exceed 0.3. The Cronbach’s alpha value for the four factors, along with the item-total correlation coefficient, mean, standard deviation, and factor loading for each item, are presented in Table 4. Upon reviewing the table, it was observed that the Cronbach’s alpha reliability coefficients for the four subdimensions ranged from 0.761 to 0.955. The average total scale score for the study group was calculated as 91.23 ± 23.10. Furthermore, the mean scores for the subdimensions were as follows: “Competence”: 42.77 ± 13.15, “Literacy”: 27.72 ± 7.22, “Opportunities”: 10.74 ± 3.91, “Barriers”: 10.00 ± 2.95.

Table 5 presents the comparison of scale scores between branches and main/subspecialty research assistants. While the median score on the scale for main specialty research assistants was 91, the median score for subspecialty research assistants was 106, which was found to be statistically significantly higher. There was also a significant difference in scale scores between branches (p = 0.024). Pairwise comparisons revealed that the difference was between basic medical sciences and surgical medical sciences. Accordingly, the scale score of basic medical science research assistants (median: 102) was significantly higher than that of surgical medical science research assistants (median: 87.5).

**Figure 1:**
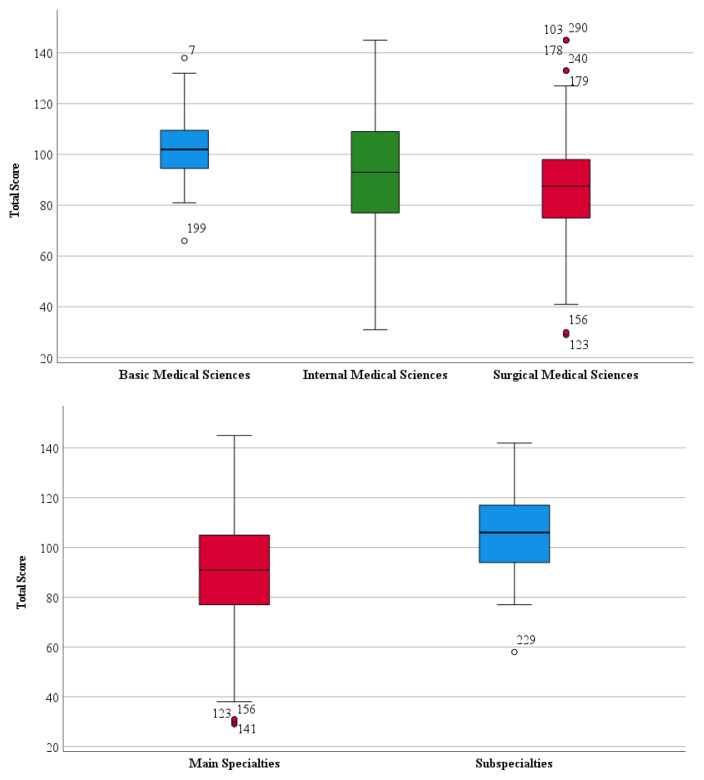
Distribution of scale scores by branches and main/subspecialties.

Figure 1 shows the distribution of scale scores by department and main/sub specialty research assistants.

The results of the ROC analysis for involvement in at least one publication or project are presented in Table 6. The area under the curve (AUC) was 0.669 (95% CI: 0.608–0.730), with a standard error of 0.031. The results of the ROC analysis for the scale scores were statistically significant (p < 0.001). The score at which the sum of sensitivity and specificity was maximized was 92.5. Therefore, a value close to this point, 92, was accepted as the cut-off point (Table 6).

**Figure 2:**
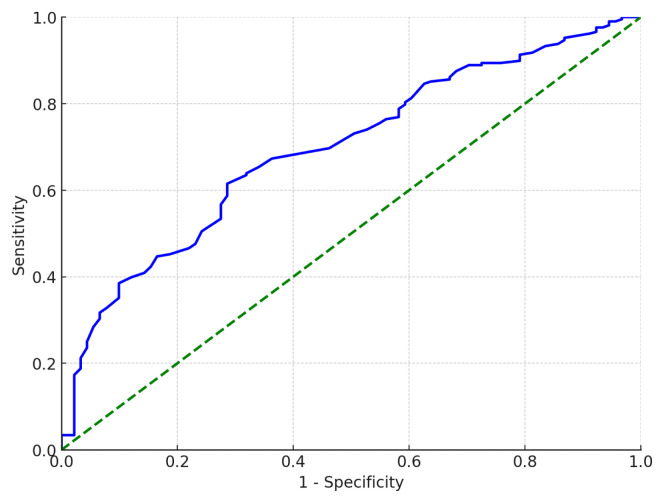
ROC curve for determining scientific competence and activity based on scale scores.

Figure 2 presents the ROC curve drawn for determining scientific competence and activity status based on the scale score.

For a scale score of 92, the calculated sensitivity was 63.2%, specificity was 65.3%, positive predictive value was 66.2%, and negative predictive value was 62.3%. It was observed that among the 299 research assistants in the study group, 148 had scores greater than 92 on the SCAS, placing them in the group considered scientifically competent and active. Meanwhile, 151 individuals had scores of 92 or below, placing them in the group considered scientifically less competent and active (Table 7).

## 4. Discussion

The mean age of the research assistants involved in this study was 28.8 years (range: 24–48), with 58.2% being women and 43.5% married. Among the participants, 25.4% had completed 0–12 months of training, 17.7% had 1–2 years, and 56.9% had more than 2 years of training. A total of 65.9% of the research assistants were from internal, 28.8% from surgical, and 5.3% from basic medical sciences. In a study by Bakioğlu et al., it was found that research assistants were equally distributed by gender, with 46% married, 60% aged 24–27 years, and 37% having 2–3 years of seniority [6]. In another study conducted with ear, nose, and throat residents, 39.6% were women, and the average age was 28.2 years (range: 25–33). The duration of training was as follows: 2 years (26.4%), 3 years (19.8%), 4 years (29.6%), and 5 years or more (24.2%) [9]. A study conducted in Konya reported that 47.5% of the research assistants were women, with an average age of 28.3 years (range: 24–41), and 50.6% were married. Similar to our findings, 63.1% were from internal, 30.7% were from surgical, and 6.2% were from basic medical sciences [20]. At Pamukkale University Faculty of Medicine, a study of specialty students found an average age of 28.3 years (range: 25–40), with 49.0% women and 38.1% married. Regarding seniority, 37.4% were in their first year, 39.7% had 13–36 months, and 22.9% had 37–60 months of training. Participants were distributed as 64.2% from internal medical sciences, 27.1% from surgical medical sciences, and 8.7% from basic medical sciences [21]. In a study conducted at İstanbul University Faculty of Medicine, the average age was 28.4 years (range: 25–39), with 59% being women. Participants were on average in their 23.5th month of specialty training, with 66% from internal, 23% from surgical, and 11% from basic medical sciences [22]. The frequency of female research assistants in our study is similar to that reported in the İstanbul University study but higher compared to other studies mentioned above. The frequency of being married aligns with the findings of Bakioğlu et al., but is lower than the study conducted in Konya. These differences may stem from the varying sociodemographic characteristics of research assistants based on city or university. The results also indicate that the distribution across branches is similar to that observed in other universities, validating our stratified sampling approach based on the number of research assistants.

Among the research assistants, 52.5% reported having sufficient foreign language proficiency to follow scientific publications. Additionally, 20.4% had attended an international congress, symposium, or workshop; domestically, 42.1% had attended three or more, and 36.5% had attended one or two. It was found that 66.9% had no original research articles, and 50.5% had no publications at all. Similarly, a study conducted at Istanbul University Faculty of Medicine found that 52% of residents could only read literature in a foreign language [22]. In the study by Bakioğlu et al., 30% of research assistants had attended four or more symposia, and 38% had no publications, a frequency lower than in our study [6]. This difference may be due to the younger age and lower seniority of research assistants in Bakioğlu’s study. Among Ear, Nose, and Throat residents, participation in one or more nonthesis research projects during residency was 74.7%, poster/oral presentation participation was 52.8%, published scientific studies were 47.3%, and research article publication was 14.3% [9]. At Pamukkale University Faculty of Medicine, 39.7% of specialty students had participated in at least one research project [21]. In a study conducted with obstetrics and gynecology residents, 61.0% stated they had participated in a research project because it was mandatory during their residency training [23]. In a medical school in India, 61.2% of postgraduate students had participated in research projects, and 31.1% had presented at national/international conferences [24]. In Japan, participation in clinical research was reported to be 68.0% [25]. At a tertiary hospital in India, only a small percentage of residents with 2–3 years of seniority had conducted research, with just 4% having published an article and 28% having presented their work at a national conference [26]. As seen, the frequency of participating in at least one research project varies greatly based on the characteristics of the groups in the conducted studies with research assistants in different institutions and specialties. The research cultures and policies of institutions or departments, along with the specialties and seniority levels of research assistants, appear to be key determining factors. Various methods can be employed to encourage specialty students to engage in research. In India, besides attending international/national conferences, it has been made mandatory to deliver oral/poster presentations and publish articles. Many developed countries motivate medical and specialty students to pursue research by supporting career development. Effective approaches may include providing mentor support from the beginning of medical school, holding meetings to discuss the importance of research, offering financial support for participation in research activities, and rewarding achievements [27–29].

In scale development studies, low correlations between items (<0.30) are interpreted as an indication that the items may not form common factors, whereas high correlations (>0.90) suggest the potential problem of multicollinearity [14,30]. Therefore, prior to factor analysis, the correlations between items were examined, and no correlation coefficients smaller than 0.30 or greater than 0.90 were observed.

One of the methods for testing construct validity in scales is exploratory factor analysis (EFA). For this analysis, the KMO test result should exceed 0.50. The KMO value indicates whether the sample size is adequate. A KMO value of 0.50–0.60 is considered poor, 0.60–0.70 weak, 0.70–0.80 moderate, 0.80–0.90 good, and greater than 0.90 excellent [14]. In this study, the KMO value was found to be 0.945. Another test used in exploratory factor analysis is Bartlett’s test of sphericity, which identifies factors at a significance level of p < 0.05. If the result of this test is p > 0.05, it indicates that the desired variance level has not been achieved, and EFA cannot be performed [31]. In this study, Bartlett’s test was significant (p < 0.001). According to the validity analysis results, the excellent KMO value and the significant p-value of Bartlett’s test of sphericity demonstrated that the correlations among the items were sufficient for conducting factor analysis. This significance indicates that the matrix formed by the relationships between variables is suitable for factor analysis [32].

In exploratory factor analysis, it is recommended to exclude items with factor loadings below 0.40 (14). However, some researchers accept a threshold value of 0.30. Items with factor loadings of 0.70 or higher are considered to explain the scale structure well [33]. If the common factor variance of an item is less than 0.10, it is highly likely that the item has an issue [34]. Using these criteria, seven items that loaded on multiple factors were removed from the scale. As a result of the principal component analysis with Varimax rotation, the scale was found to consist of 4 factors and 29 items. The factor loadings of the remaining 29 items ranged between 0.558 and 0.834. In exploratory factor analysis, it is stated that each subdimension should contain at least three items. After determining the relevant items, the factors should be named appropriately. Considering the theoretical framework, the common characteristics of the items, and the meanings expressed by items with high factor loadings [14], the scale was structured into four subdimensions, each containing at least three items, and the factors were named appropriately.

In exploratory factor analysis, it is important to determine whether a scale is unidimensional or multidimensional [35]. For unidimensional scales, the total explained variance should be at least 30%. For multidimensional scales, like ours, this amount needs to be higher [36]. In multidimensional designs, a total explained variance between 40%–60% is generally considered sufficient [34]. In this study, 4 factors explained 68.0% of the total variance. Considering these results, it can be inferred that the total explained variance of the scale is sufficient, the construct validity of the scale is established, and the items are adequately related to the scale.

In scale development studies, internal consistency is a significant indicator of reliability. For scales containing Likert-type items, Cronbach’s alpha coefficient is a measure of internal consistency and ranges between 0 and 1. Cronbach’s alpha indicates the consistency of items within the scale [37]. A Cronbach’s alpha coefficient between 0.60–0.80 suggests the scale is fairly reliable, while a value above 0.80 indicates high reliability [38,39].

In this study, the Cronbach’s alpha reliability coefficients ranged from 0.761 to 0.955 across subscales and were 0.960 for the overall scale. These Cronbach’s alpha values demonstrate that the scale is highly reliable [14]. The high Cronbach’s alpha coefficient for the final set of items suggests that the items are consistent with one another and measure nearly identical characteristics [40]. Another method to assess the reliability of a scale is by examining item-total score correlations. An item-total correlation coefficient below 0.30 suggests a potential issue with the item, and such items may be removed from the scale. Before removing items below this threshold, their impact on the Cronbach’s alpha coefficient is also considered [33]. In this study, none of the items had an item-total correlation coefficient below 0.30.

The area under the ROC curve (AUC) indicates how accurately two different groups can be distinguished. The closer the AUC value is to 1, the better the discrimination [41]. According to Hosmer et al., an AUC value of 0.7 ≤ AUC < 0.8 is interpreted as having “acceptable” discrimination [42]. In this study, the AUC was determined to be 0.669 (95% CI: 0.608–0.730). Based on the determined cut-off point (92 points), the scale’s sensitivity was 63.2%, and specificity was 65.3%.

A key limitation of this study is that the sample consisted solely of research assistants from a single medical faculty. Another significant limitation is the lack of known-groups validity analyses and group difference comparisons. Additionally, test-retest analyses to demonstrate the form’s stability over time were not performed. As a strength of the study, reaching specialty students working in every department of the faculty can be highlighted.

## 5. Conclusion

As part of the Scientific Competence and Activity Scale (SCAS) development study, it was determined that the scale items adequately represent the targeted domain (content validity), can effectively distinguish between “competent” and “active” individuals and those who are not (item-total correlation), consist of 29 items within four subdimensions based on exploratory factor analysis (construct validity), and exhibit high internal consistency (reliability analyses). The scale uses a 5-point Likert rating system (1-strongly disagree, 2-disagree, 3-neutral, 4-agree, 5-strongly agree). There are no reverse-scored items in the scale. The minimum score obtainable on the scale is 29, and the maximum is 145. The cut-off point for the scale is 92, where scores of 92 or below indicate individuals who are “not competent and active” in terms of scientific research competence and activity, while scores above 92 indicate individuals who are “competent and active”.

In conclusion, the developed Scientific Competence and Activity Scale is a valid and reliable tool that can be used with research assistants in medical faculties. Testing the theoretical structure of the scale on different sample groups is important to generalize its reliability and validity. Thus, it is advised that the reliability and validity study of the scale be repeated with different samples.

## Figures and Tables

**Table 1:** Sociodemographic and scientific activity status of participants by branch.

		Basic Medical Sciences	Internal Medical Sciences	Surgical Medical Sciences	Main specialty	Sub-specialty

		n (%)	n (%)	n (%)	n (%)	n (%)

Age	Under 30	10 (62.5)	136 (69.0)	63 (74.1)	208 (77.3)	1 (3.4)
	30 or above	6 (37.5)	61 (31.0)	22 (25.9)	61 (22.7)	28 (96.6)

	p*	0.548			**<0.001**	

Gender	Female	11 (68.8)	122 (61.9)	41 (47.7)	157 (58.1)	17 (58.6)
	Male	5 (31.3)	75 (38.1)	45 (52.3)	113 (41.9)	12 (41.4)

	p*	0.056			0.961	

Marital status	Married	10 (62.5)	93 (47.2)	27(31.4)	108 (40.0)	22 (75.9)
	Single	6 (37.5)	104 (52.8)	59(68.6)	162 (60.0)	7 (24.1)

	p*	**0.014**			**<0.001**	

Having children	Yes	2 (12.5)	24 (12.2)	9 (10.5)	21 (7.8)	14 (48.3)
	No	14 (87.5)	173 (87.8)	77 (89.5)	249 (92.2)	15 (51.7)

	p*	0.913			**<0.001**	

Duration of residency	0–12 months	7 (43.8)	48 (24.4)	21 (24.4)	67 (24.8)	9 (31.0)
	1–2 years	2 (12.5)	32 (16.2)	19 (22.1)	47 (17.4)	6 (20.7)
	More than 2 years	7 (43.8)	117 (59.4)	46 (53.5)	156 (57.8)	14 (48.3)

	p*	0.339			0.615	

Original article	Yes	5 (31.3)	65 (33.0)	29 (33.7)	72 (26.7)	27 (93.1)
	No	11 (68.8)	132 (67.0)	57 (66.3)	198 (73.3)	2 (6.9)

	p*	0.980			**<0.001**	

Case report/series	Yes	5 (31.3)	53 (25.9)	28 (32.6)	71 (26.3)	15 (51.7)
	No	11 (68.8)	144 (73.1)	58 (67.4)	199 (73.7)	14 (48.3)

	p*	0.611			**0.004**	

Traditional review	Yes	1 (6.3)	30 (15.2)	8 (9.3)	35 (13.0)	4 (13.8)
	No	15 (93.8)	167 (84.8)	78 (90.7)	235 (87.0)	25 (86.2)

	p*	0.281			0.779	

Systematic review/meta-analysis	Yes	1 (6.3)	5 (2.5)	5 (5.8)	10 (3.7)	1 (3.4)
	No	15 (93.8)	192(97.5)	81(94.2)	260 (96.3)	28 (96.6)

	p*	0.345			0.945	

Book/Book Chapter	Yes	2 (12.5)	31 (15.7)	19(22.1)	35 (13.0)	17 (58.6)
	No	14 (87.5)	166(84.3)	67(77.9)	235 (87.0)	12 (41.4)

	p*	0.374			**<0.001**	

Poster presentation	0	7 (43.8)	116 (58.9)	48 (55.8)	167 (61.9)	4 (13.8)
	1	3 (18.8)	37 (18.8)	10 (11.6)	48 (17.8)	2 (6.9)
	2 or more	6 (37.5)	44 (22.3)	28 (32.6)	55 (20.4)	23 (79.3)

	p*	0.219			**<0.001**	

Oral presentation	0	8 (50.0)	132 (67.0)	44 (51.2)	181 (67.0)	3 (10.3)
	1	4 (25.0)	43 (21.8)	17 (19.8)	53 (19.6)	11 (37.9)
	2 or more	4 (25.0)	22 (11.2)	25 (29.1)	36 (13.3)	15 (51.7)

	p*	**0.005**			**<0.001**	

National scientific project	Yes	3 (18.8)	21 (10.7)	15 (17.4)	29 (10.7)	10 (34.5)
	No	13 (81.3)	176 (89.3)	71 (82.6)	241 (89.3)	19 (65.5)

	p*	0.233			**<0.001**	

International scientific project	Yes	0 (0.0)	3 (1.5)	5 (5.8)	5 (1.9)	3 (10.3)
	No	16 (100.0)	194 (98.5)	81 (94.2)	265 (98.1)	26 (89.7)

	p*	0.095			**0.033**	

National congress, symposium etc.	0	2 (12.5)	48 (24.4)	14(16.3)	62 (23.0)	2 (6.9)
	1–2	7 (43.8)	67 (34.0)	35 (40.7)	105 (38.9)	4 (13.8)
	3 or more	7 (43.8)	82 (41.6)	37 (43.0)	103 (38.1)	23 (79.3)

	p*	0.482			**<0.001**	

International congress, symposium etc.	Yes	5 (31.3)	32 (16.2)	24 (27.9)	47 (17.4)	14 (48.3)
	No	11 (68.8)	165 (83.8)	62 (72.1)	223 (82.6)	15 (51.7)

	p*	**0.044**			**<0.001**	

Presence of scientific activity in the department	Weekly or more	14 (87.5)	147 (75.8)	63 (75.9)	202 (75.9)	22 (81.5)
	Every 15 days/monthly	2 (12.5)	28 (14.4)	11 (13.3)	39 (14.7)	2 (7.4)
	Irregular or never	0 (0.0)	19 (9.8)	9 (10.8)	25 (9.4)	3 (11.1)

	p*	0.728			0.578	

Quality of scientific activity in the department	Sufficient	15 (93.8)	98 (50.5)	33 (40.2)	127 (47.9)	19 (70.4)
	Partially sufficient	1 (6.3)	61 (31.4)	26 (31.7)	82 (30.9)	6 (22.2)
	Insufficient	0 (0.0)	35 (18.0)	23 (28.0)	56 (21.1)	2 (7.4)

	p*	**0.002**			0.067	

n: Frequency, %: Column percentage,

* Chi-square tests

**Table 2:** Results of the Kaiser-Meyer-Olkin (KMO), Bartlett’s Test of Sphericity, and principal component analysis of the scale.

Kaiser-Meyer-Olkin (KMO) Value		0.945

Bartlett’s Test of Sphericity Values	Chi-square value	7387.1
	p-value	<0.001

**Item**		
16	I am able to identify journals that are appropriate for the topic of my study.	
18	I am capable of analyzing tables and graphs presented in scientific publications.	
19	I can present the findings of my research in the form of tables and figures.	
26	I am able to write my research as a manuscript in compliance with academic standards.	
27	I possess the necessary resources (e.g., facilities, personnel, patients, laboratory) to engage in scientific activities.	
33	I consider myself sufficiently competent to conduct scientific research.	
34	I do not have mental health issues that would hinder my ability to conduct scientific research.	

**Table 3:** Factor loadings of the items comprising the scale.

No	Item	Competence	Literacy	Opportunities	Barriers
I1	I am capable of determining the appropriate research methodology aligned with the objectives of a scientific study.	0.592			
I2	I know the differences and subtypes of observational, experimental, and methodological research designs.	0.708			
I3	I know the levels of the evidence pyramid.	0.733			
I4	I know the research guidelines such as STROBE, CONSORT, PRISMA.	0.705			
I5	I am aware of the ethical principles I must adhere to when conducting research, such as the Declaration of Helsinki.	0.721			
I6	I know the concepts of bias and confounding variables.	0.754			
I7	I am capable of selecting a sample that accurately represents the study population.	0.755			
I8	I can define the dependent and independent variables of a study.	0.768			
I9	I am aware of the sources for locating the index and Q rankings of journals.	0.712			
I10	I am capable of randomly selecting the groups to be included in the research.	0.745			
I11	I am proficient in using suitable statistical programs to analyze data.	0.694			
I12	I am knowledgeable about the meaning of p-value and confidence intervals.	0.686			
I13	I am capable of preparing the study results in a publishable format.	0.708			
I14	I am knowledgeable about citation standards, such as APA and Vancouver formats	0.709			
I15	I am capable of formulating research topics and hypotheses in my area of expertise.		0.619		
I16	I have sufficient proficiency in a foreign language to follow scientific publications.		0.558		
I17	I keep up with current developments in the field of science.		0.723		
I18	I use textbooks to obtain accurate and reliable knowledge.		0.661		
I19	I have a strong desire to stay informed about scientific developments.		0.738		
I20	In addressing problems encountered in my work environment, I primarily refer to scientific sources.		0.768		
I21	I actively read a sufficient number of scientific studies in my discipline.		0.744		
I22	I conduct sufficient searches on platforms such as PubMed, Google Scholar, and UpToDate within my field.		0.799		
I23	I have sufficient time for scientific activities.			0.834	
I24	I am able to find the necessary financial resources for my research.			0.762	
I25	I do not have a workload or commitments that would hinder my research.			0.777	
I26	I have sufficient motivation to conduct scientific research.			0.617	
I27	I do not experience mobbing in my work environment that would interfere with my scientific work.				0.682
I28	Personal financial concerns do not hinder my ability to conduct scientific research.				0.583
I29	My family responsibilities do not interfere with my scientific work.				0.704

**Table 4:** Factor and item analysis results of the scale.

		Mean ± SD	Item-total correlation	Cronbach-alpha

**Scientific competence and activity scale**		**91.23 ± 23.10**	**-**	**0.960**

**Factor 1 (Competence)**	I1	3.30 ± 1.09	0.733	0.955
	I2	3.11 ± 1.12	0.771	
	I3	3.14 ± 1.22	0.689	
	I4	2.48 ± 1.13	0.644	
	I5	3.27 ± 1.20	0.752	
	I6	3.07 ± 1.21	0.698	
	I7	2.96 ± 1.16	0.740	
	I8	3.22 ± 1.14	0.762	
	I9	2.92 ± 1.28	0.710	
	I10	3.07 ± 1.17	0.775	
	I11	2.57 ± 1.24	0.611	
	I12	3.43 ± 1.21	0.736	
	I13	3.08 ± 1.15	0.752	
	I14	3.14 ± 1.24	0.744	
	**Factor 1 Total Score**	42.77 ± 13.15	-	

**Factor 2 (Literacy)**	I15	3.52 ± 1.06	0.702	0.937
	I16	3.43 ± 1.16	0.659	
	I17	3.27 ± 1.09	0.786	
	I18	3.65 ± 1.06	0.655	
	I19	3.61 ± 1.07	0.699	
	I20	3.59 ± 1.09	0.735	
	I21	3.12 ± 1.03	0.763	
	I22	3.53 ± 1.11	0.672	
	**Factor 2 Total Score**	27.72 ± 7.22	-	

**Factor 3 (Opportunities)**	I23	2.73 ± 1.24	0.494	0.835
	I24	2.49 ± 1.09	0.413	
	I25	2.65 ± 1.29	0.396	
	I26	2.87 ± 1.16	0.622	
	**Factor 3 Total Score**	10.74 ± 3.91	-	

**Factor 4 (Barriers)**	I27	3.43 ± 1.18	0.524	0.761
	I28	3.23 ± 1.19	0.509	
	I29	3.33 ± 1.21	0.472	
	**Factor 4 Total Score**	10.0 ± 2.95	-	

SD: Standard Deviation

**Table 5:** Comparison of scale scores between departments and primary/subspecialties.

	n	Mean ± SD	Median	Min–Max	p

Main specialty	270	89.55 ± 22.89	91.00	29–145	**<0.001** *
Subspecialty	29	106.86 ± 19.08	106.00	58–142	

Basic medical sciences	16	102.06 ± 17.38	102.00	66–138	**0.024** **
Internal medical sciences	197	91.96 ± 22.92	93.00	31–145	
Surgical medical sciences	86	87.55 ± 23.85	87.50	29–145	

Total	299	91.23 ± 23.10	92.00	29–145	

SD: Standard Deviation,

* Mann-Whitney U test,

** Kruskal-Wallis test

**Table 6:** Sensitivity and specificity values of total scale scores for participation in at least one publication or project.

Scale score	Sensitivity	Specificity	Sensitivity + Specificity
88.50	0.684	0.576	1.260
89.50	0.671	0.597	1.268
90.50	0.671	0.604	1.275
91.50	0.645	0.625	1.270
92.50	0.632	0.653	1.285
93.50	0.613	0.667	1.280
94.50	0.574	0.674	1.248
95.50	0.555	0.715	1.270
96.50	0.529	0.736	1.265

* Multiply by 100.

**Table 7:** Accuracy values of the scale for a cut-off score of 92.

Scientific competence and activity	Participation in at least one publication or project		Total
	Yes n (%)	No n (%)	n (%)
Competent (Above 92 points)	98 (32.8)	50 (16.7)	148 (49.5)
Not Competent (92 points or below)	57 (19.1)	94 (31.4)	151 (50.5)
Total	155 (51.8)	144 (48.2)	299 (100.0)

n: Frequency, %: Percentage
